# Reanalysis of public bulk and single-nucleus transcriptomes identifies widespread tumor-normal dysregulation and a cross-layer cell-cycle signal in sinonasal squamous cell carcinoma

**DOI:** 10.3389/fgene.2026.1918021

**Published:** 2026-10-22

**Authors:** Jiongcong Yuan, Ying Wang, Kaisheng Lin, Lingli Sun

**Affiliations:** 1 Department of Otolaryngology, Jieyang People’s Hospital, Jieyang, China; 2 Department of Otorhinolaryngology, The Fourth Affiliated Hospital of Soochow University, Suzhou, China

**Keywords:** CCL5, CD74, human papillomavirus, IL32, immune microenvironment, immunotherapy biomarkers, single-cell RNA sequencing, sinonasal squamous cell carcinoma

## Abstract

**Background:**

Sinonasal squamous cell carcinoma (SNSCC) is a rare malignancy affecting the nasal cavity and paranasal sinuses, with human papillomavirus (HPV) infection identified as a critical etiological factor in a subset of cases. HPV-positive and HPV-negative SNSCC exhibit distinct clinical behaviors, with HPV-positive tumors generally demonstrating better prognosis and treatment response. However, the cellular composition and transcriptomic landscape of SNSCC tumor versus normal tissue remain incompletely characterized. Multi-layer transcriptomic reanalysis of publicly available single-nucleus and bulk RNA sequencing data provides an opportunity to systematically identify tumor-normal gene expression changes and nominate candidate biomarkers for prospective validation.

**Methods:**

We reanalyzed publicly available single-nucleus RNA sequencing data (GSE277797: one healthy nasal mucosa library, HC1; one SNSCC tumor library, CA5) together with bulk RNA-seq data from 14 SNSCC and normal nasal mucosa samples (GSE278145), microarray data from five matched tumor-adjacent-normal pairs (GSE138809), and a 21-sample sinonasal cancer tumor-only context cohort (GSE118386). Following rigorous quality control, doublet detection, cell-type annotation using canonical markers, and program scoring, we integrated findings across data layers. Candidate genes were further assessed by *in vitro* experiments in two SNSCC cell lines (RPMI 2650 and Detroit 562) compared with normal bronchial epithelial BEAS-2B cells, using quantitative real-time PCR (qRT-PCR) to evaluate mRNA expression levels (2^−ΔΔCT^ method, GAPDH reference) and enzyme-linked immunosorbent assay (ELISA) to quantify secreted and intracellular protein concentrations.

**Results:**

Single-nucleus analysis resolved 14,479 nuclei into 11 biological cell types, with the largest observed compositional shift being an increase in Epithelial cells (+44.7 percentage points) and a decrease in Secretory epithelial cells (−56.2 percentage points) in the tumor library relative to the healthy library. Bulk RNA-seq differential expression testing across 17,677 genes identified 5,732 genes at FDR <0.05, with cell-cycle genes showing the largest program-level shift (+1.727 standardized units). Cross-layer integration yielded a Spearman correlation of 0.543 between bulk fold-changes and single-nucleus pseudobulk shifts. Candidate genes including CD74, CCL5, IL32, and HLA-DRA showed concordant upregulation across data layers. *In vitro* qRT-PCR confirmed significant mRNA upregulation of CD74 (4.27-fold), CCL5 (3.15-fold), IL32 (3.68-fold), and HLA-DRA (3.82-fold) in RPMI 2650 cells compared to normal BEAS-2B cells (all p < 0.01), with elevated protein levels further corroborated by enzyme-linked immunosorbent assay.

**Conclusion:**

This multi-layer transcriptomic reanalysis of publicly available SNSCC datasets identifies consistent tumor-versus-normal dysregulation across single-nucleus and bulk platforms, and nominates CD74, CCL5, IL32, and HLA-DRA as candidate biomarkers warranting prospective clinical validation. These findings are exploratory and should be interpreted within the constraints of the available single-sample single-nucleus data.

## 1 Introduction

Sinonasal squamous cell carcinoma (SNSCC) is an uncommon but clinically significant malignancy arising from the epithelial lining of the nasal cavity and paranasal sinuses, accounting for approximately 3%–5% of all head and neck cancers (Llorente et al., 2014; Dutta et al., 2015). Despite advances in multimodality treatment approaches including surgery, radiotherapy, and chemotherapy, SNSCC continues to present substantial challenges due to its anatomically complex location, tendency for local invasion, and historically poor prognosis with 5-year survival rates ranging from 30% to 50% (Lund et al., 2010; Amit et al., 2019). The anatomic constraints and proximity to critical structures often preclude complete surgical resection, necessitating aggressive adjuvant therapies that carry significant morbidity.

Human papillomavirus (HPV) infection has emerged as a crucial etiological and prognostic factor in a subset of SNSCC cases, with HPV-positive tumors comprising approximately 20%–50% of sinonasal malignancies depending on anatomic subsite and geographic location (Bishop et al., 2013; Larque et al., 2014). Paralleling observations in oropharyngeal squamous cell carcinoma, HPV-positive SNSCC demonstrates markedly different clinical behavior compared to HPV-negative disease, including younger patient age at presentation, better response to radiotherapy and chemotherapy, and significantly improved overall survival (Alos et al., 2009; Wang et al., 2025; Laco et al., 2015). Notably, the identification of reliable molecular biomarkers that can distinguish HPV-driven from HPV-independent disease represents an unmet clinical need; parallel efforts in other inflammatory disease contexts have demonstrated that novel circulating and transcriptomic markers identified through multi-platform approaches can substantially refine risk stratification and guide therapeutic decisions (Wang et al., 2025). These clinical distinctions suggest fundamental biological differences in tumor pathogenesis, immune evasion mechanisms, and therapeutic vulnerabilities between HPV-driven and HPV-independent SNSCC, though the molecular basis of these differences requires investigation in appropriately powered multi-sample studies.

The tumor immune microenvironment plays a critical role in cancer progression, treatment response, and clinical outcomes (Hanahan and Weinberg, 2011). HPV-positive tumors are hypothesized to harbor distinct immune microenvironments characterized by enhanced immune cell infiltration, active antitumor immunity, and altered checkpoint expression profiles compared to HPV-negative counterparts (Feng et al., 2017). Viral antigen presentation by HPV-infected cells may trigger robust adaptive immune responses, while viral oncoproteins E6 and E7 can modulate host immune signaling pathways (Münger et al., 2004). Understanding tumor-versus-normal transcriptomic changes in SNSCC is particularly relevant given the recent success of immune checkpoint inhibitors in treating recurrent or metastatic head and neck squamous cell carcinoma (Ferris et al., 2016), yet candidate biomarkers for immunotherapy response in SNSCC remain poorly defined.

Traditional bulk RNA sequencing and histopathological analyses provide averaged measurements across heterogeneous cell populations, obscuring critical cellular subpopulations and cell-cell interactions that drive tumor behavior (Tang et al., 2009). Single-cell RNA sequencing (scRNA-seq) has revolutionized cancer biology by enabling high-resolution dissection of tumor ecosystems at individual cell resolution, revealing previously unrecognized cellular states, rare cell populations, and complex intercellular communication networks (Papalexi and Satija, 2018; Thrane et al., 2018). In head and neck cancers, scRNA-seq studies have identified novel cancer-associated fibroblast subsets, characterized exhausted T cell populations, and mapped tumor-infiltrating immune cell heterogeneity (Puram et al., 2017; Cillo et al., 2020).

Despite the clinical importance of HPV status in SNSCC and the transformative potential of immunotherapy, comprehensive transcriptomic characterization of tumor-versus-normal remodeling in SNSCC using multi-layer public data has not been systematically reported. Such investigation could identify candidate immunotherapy-relevant genes, characterize cellular composition shifts between healthy and tumor tissue, and generate hypotheses for prospective validation. Key questions addressed descriptively here include: What cellular composition shifts are observed between one healthy and one SNSCC single-nucleus library? Which bulk-level genes show consistent tumor-versus-normal differential expression? Which candidates show cross-layer concordance?

In this study, we reanalyzed publicly available transcriptomic data from NCBI GEO (accessions GSE277797, GSE278145, GSE138809, and GSE118386) to investigate SNSCC tumor-versus-normal gene expression remodeling. Through integrative computational analysis spanning single-nucleus and bulk transcriptomic layers, we aimed to: (1) characterize cellular composition and program activity across one healthy and one SNSCC single-nucleus library; (2) identify bulk-level tumor-versus-normal differentially expressed genes across 14 samples; (3) assess cross-layer concordance between single-nucleus and bulk observations; (4) nominate candidate genes with tumor-versus-normal upregulation for preliminary *in vitro* assessment. Our findings provide preliminary descriptive insights into tumor-versus-normal transcriptomic remodeling in SNSCC and nominate candidate genes for prospective validation in larger independent cohorts.

## 2 Methods

### 2.1 Data sources and disease scope

The reanalysis was defined around sinonasal squamous cell carcinoma (SNSCC) and used the supplied public accession files without replacing missing metadata with proxy cohorts. From the broader GSE277797 snMultiome series, the analyzed RNA subset comprised GSM8530826 (HC1 healthy nasal mucosa) and GSM8530830 (CA5 SNSCC tumor); only the gene-expression layer was used. GSE278145 supplied raw bulk RNA-seq counts for 7 SNSCC tumors and seven normal nasal mucosa samples. GSE138809 supplied five matched SNSCC tumor and adjacent-normal microarray pairs. The analyzed 21-sample NKSCC subset of the broader GSE118386 sinonasal-cancer series supplied tumor-only subtype context. No TCGA dataset was used because a same-disease TCGA SNSCC transcriptome or single-cell cohort was not available in the supplied sources; TCGA head-and-neck cohorts were not substituted. The available data do not support an HPV-positive versus HPV-negative comparison, so no HPV claim is made.

### 2.2 Single-nucleus processing and quality control

The two 10x H5 matrices were read as sparse count matrices, concatenated after gene-symbol harmonization, and retained as a merged object in Data/processed_adata.h5ad with raw counts in the counts layer. Per library, lower cutoffs were the empirical fifth percentiles for detected genes and total counts; upper cutoffs were the empirical 99.5th percentiles for total counts, detected genes, and mitochondrial fraction. This library-wise rule was used to preserve the distinct depth distributions rather than imposing a pooled threshold. Doublets were flagged with scDblFinder per library. Ambient RNA was not estimated because empty-droplet matrices were not supplied. The final object retained 14,479 nuclei across 36,601 genes; HC1 retained 6,817 nuclei and CA5 retained 7,662.

### 2.3 Normalization, embedding, annotation, and program scores

Counts were library-size normalized and log1p transformed for visualization and score calculation. Highly variable genes, PCA, a neighborhood graph, and UMAP were computed with fixed seeds; Leiden communities were used only as an internal aid and are not exposed as biological claims. Cell labels were assigned from observed marker programs and refined against canonical epithelial, stromal, vascular, myeloid, lymphoid, plasma, ciliated, and tuft markers. The final biological labels were: Myeloid cells, Secretory epithelial cells, Perivascular cells, Ciliated epithelial cells, Plasma cells, T and NK cells, Epithelial cells, Endothelial cells, Fibroblasts, Basal epithelial cells, Tuft cells. Program scores were calculated as the mean normalized expression of pre-specified genes in Data/gene_sets.tsv. UMAPs, composition summaries, and program comparisons are descriptive because each condition contributes one library rather than independent patients.

### 2.4 Bulk RNA-seq differential expression

GSE278145 raw counts were filtered for minimal expression, TMM-normalized, and modeled with edgeR robust quasi-likelihood testing using design ∼ condition and the tumor-minus-normal contrast. The analysis tested 17,677 genes across 14 libraries (seven tumor and seven normal). Benjamini–Hochberg FDR <0.05 defined the reported differential set. This is a sample-level comparison across the analyzed libraries; the source metadata do not establish pairing or provide HPV status, recurrence, batch, or outcome covariates.

### 2.5 Independent microarray context

For GSE138809, the five tumor-adjacent-normal pairs were analyzed with a paired limma model containing pair and condition terms. Because the platform annotation did not provide reliable genome-wide gene-symbol mapping, results remain at probe level and probe identifiers are preserved in Figure 7. The analyzed 21-sample NKSCC subset of GSE118386 was processed as tumor-only expression context; its expression matrix and pre-specified program scores are shown descriptively and are not used as tumor-normal validation.

### 2.6 Cross-layer integration and statistical boundaries

Bulk edgeR effects were matched to single-nucleus pseudobulk shifts by exact gene symbol. Candidate panels were restricted to pre-specified genes with sufficient measured expression, a bulk FDR threshold, and concordant observed direction where stated. The single-nucleus layer contributes one healthy and one tumor library, so cell-level observations are not treated as independent patient replicates and no single-cell group P values are reported. External tumor-only panels are described as context, not replication.

### 2.7 In vitro cell culture, qRT-PCR, and ELISA

Two SNSCC cell lines (RPMI 2650 and Detroit 562) and the normal bronchial epithelial cell line BEAS-2B were obtained from the American Type Culture Collection (ATCC, Manassas, VA, United States of America). RPMI 2650 and Detroit 562 cells were cultured in RPMI-1640 medium (Gibco, Thermo Fisher Scientific, Waltham, MA, United States of America) supplemented with 10% fetal bovine serum (FBS; Gibco) and 1% penicillin-streptomycin (Gibco). BEAS-2B cells were maintained in Bronchial Epithelial Cell Growth Medium (BEGM; Lonza, Basel, Switzerland) according to the manufacturer’s instructions. All cell lines were incubated at 37 °C in a humidified atmosphere containing 5% CO2. For qRT-PCR, total RNA was extracted from approximately 1 × 10^6^ cells per line using TRIzol reagent (Invitrogen, Thermo Fisher Scientific) following the manufacturer’s protocol. RNA concentration and purity were assessed by NanoDrop 2000 spectrophotometry (Thermo Fisher Scientific); samples with A260/A280 ratios between 1.8 and 2.1 were used. Complementary DNA (cDNA) was synthesized from 1 μg of total RNA using the PrimeScript RT Reagent Kit with gDNA Eraser (TaKaRa Bio, Kusatsu, Japan) according to the manufacturer’s instructions. Quantitative real-time PCR (qRT-PCR) was performed on a LightCycler 480 system (Roche Diagnostics, Basel, Switzerland) using TB Green Premix Ex Taq II (TaKaRa Bio). Amplification conditions were: 95 °C for 30 s; 40 cycles of 95 °C for 5 s and 60 °C for 30 s; followed by melt-curve analysis to confirm amplicon specificity. Each reaction was run in triplicate. Relative mRNA expression was calculated using the 2^−ΔΔCT^ method with GAPDH as the housekeeping reference gene. Primer sequences used were: CD74 forward 5′-AGC​ACC​AGC​AAG​ATC​ACA​GAG-3′, reverse 5′-TTC​AGG​TCC​TCA​AAG​GTG​AGC-3′; CCL5 forward 5′-CTG​CTT​TGC​CTA​CAT​TGC​CCG-3′, reverse 5′-TCG​GGT​GAC​AAA​GAC​GAC​TG-3′; IL32 forward 5′-GAA​GAT​CCA​GAG​CCT​TCA​GCA-3′, reverse 5′-CAG​TAG​TTT​CCA​AGC​CGC​TG-3′; HLA-DRA forward 5′-TGG​AGC​GTC​TGC​GCT​ATG​AC-3′, reverse 5′-AGC​TGT​GTG​TTC​CTG​CAG​GT-3′; GAPDH forward 5′-GGA​GCG​AGA​TCC​CTC​CAA​AAT-3′, reverse 5′-GGC​TGT​TGT​CAT​ACT​TCT​CAT​GG-3′. For ELISA, cells were seeded in 6-well plates and cultured to 80%–90% confluency. Culture supernatants were collected after 48 h and centrifuged at 1,000×g for 10 min to remove cell debris; intracellular protein fractions were prepared by lysing cell pellets in RIPA buffer (Beyotime Biotechnology, Shanghai, China) supplemented with protease inhibitor cocktail (Roche) on ice for 30 min, followed by centrifugation at 12,000×g for 15 min at 4 °C. Total protein concentration was determined by BCA assay (Thermo Fisher Scientific). Protein levels of CD74, CCL5, IL32, and HLA-DRA were quantified using commercially available enzyme-linked immunosorbent assay (ELISA) kits (Cloud-Clone Corp., Wuhan, China) according to the manufacturer’s protocols; absorbance was measured at 450 nm on a microplate reader (BioTek Synergy HTX, Agilent, Santa Clara, CA, United States of America). All ELISA measurements were performed in triplicate. Statistical comparisons between SNSCC cell lines and BEAS-2B were performed using an unpaired two-tailed Student’s t-test; p < 0.05 was considered statistically significant. All *in vitro* experiments were conducted in at least three independent biological replicates.

## 3 Results

### 3.1 Input QC and library-scale data audit

Detected-gene distributions are shown for HC1 and CA5 with empirical lower cutoffs of 427 and 469 genes per barcode, respectively (Figure 1A). The basic-QC barcode set contained 16,486 of 17,555 input barcodes; hexagonal bins aggregate observed barcode-level UMI and gene counts, with color encoding log10 density (Figure 1B). Per-library mitochondrial upper limits were the empirical 99.5th percentiles: HC1 4.56% and CA5 3.70% (Figure 1C). scDblFinder score distributions are shown for the 7,675 HC1 and 8,811 CA5 barcodes that passed basic QC; 858 and 1,149, respectively, were flagged (Figure 1D). Filtering trajectories show input, basic-QC, and final retained counts: HC1 8,140, 7,675, and 6,817; CA5 9,415, 8,811, and 7,662. Final counts exclude flagged doublets (Figure 1E). GSE278145 contributed 14 bulk libraries (7 normal and seven tumor); raw library sizes range from 413,778,112 to 716,711,739 reads (Figure 1F).

**FIGURE 1:**
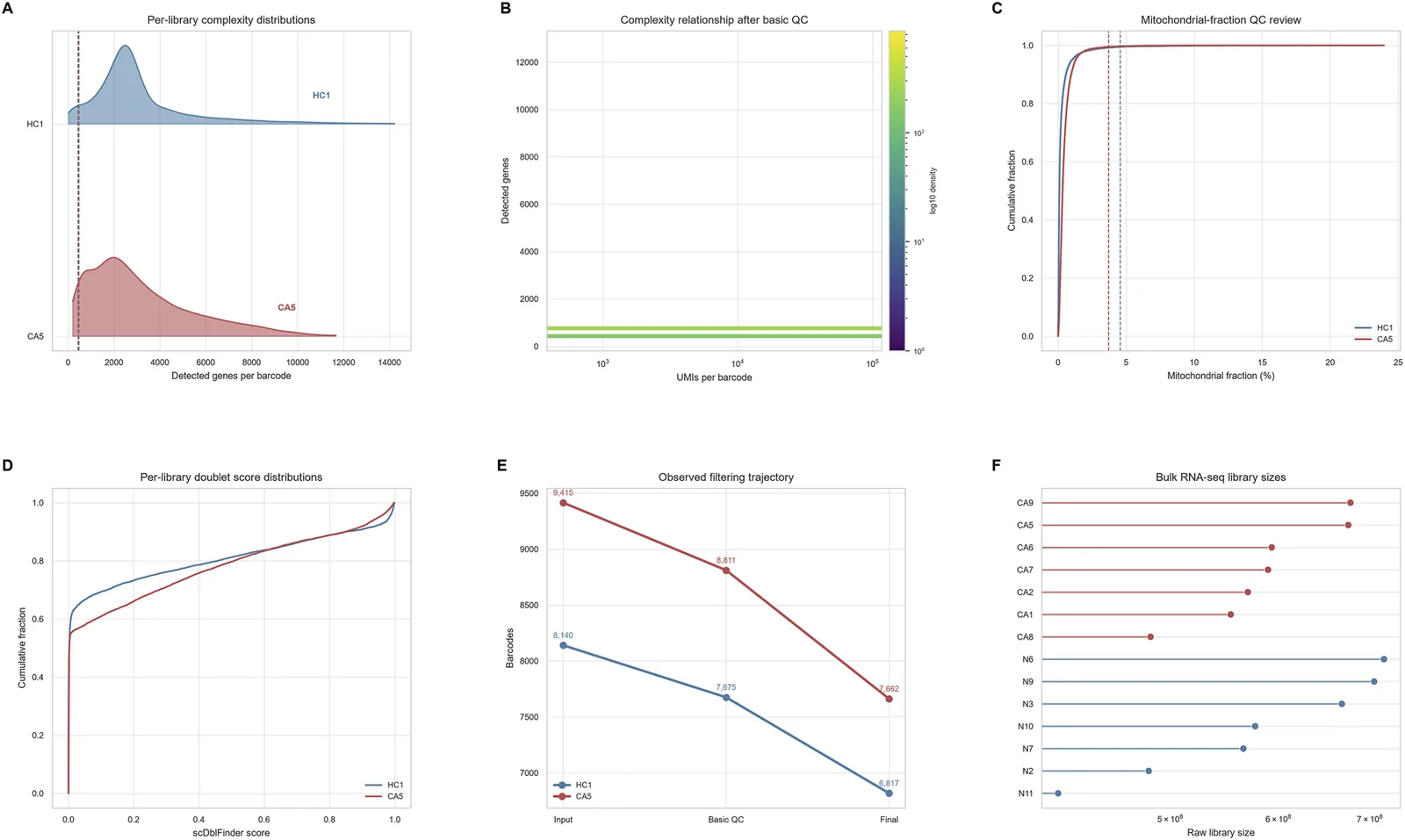
Input QC and library-scale data audit. **(A)** Detected-gene distributions are shown for HC1 and CA5 with empirical lower cutoffs of 427 and 469 genes per barcode, respectively. **(B)** The basic-QC barcode set contained 16,486 of 17,555 input barcodes; hexagonal bins aggregate observed barcode-level UMI and gene counts, with color encoding log10 density. **(C)** Per-library mitochondrial upper limits were the empirical 99.5th percentiles: HC1 4.56% and CA5 3.70%. **(D)** scDblFinder score distributions are shown for the 7,675 HC1 and 8,811 CA5 barcodes that passed basic QC; 858 and 1,149, respectively, were flagged. **(E)** Filtering trajectories show input, basic-QC, and final retained counts: HC1 8,140, 7,675, and 6,817; CA5 9,415, 8,811, and 7,662. Final counts exclude flagged doublets. **(F)** GSE278145 contributed 14 bulk libraries (7 normal and seven tumor); raw library sizes range from 413,778,112 to 716,711,739 reads.

### 3.2 Merged single-nucleus cellular atlas

The merged UMAP contains 14,479 retained nuclei annotated into 11 biological cell types: Epithelial cells, Secretory epithelial cells, Fibroblasts, Perivascular cells, T and NK cells, Endothelial cells, Myeloid cells, Ciliated epithelial cells, Basal epithelial cells, Tuft cells, Plasma cells (Figure 2A). HC1 contributes 6,817 healthy nuclei and CA5 contributes 7,662 tumor nuclei; this panel displays library provenance rather than a patient-level comparison (Figure 2B). The dot matrix uses observed normalized expression and detection fractions for 10 canonical genes across 11 labeled cell types (Figure 2C). The highest healthy fractions are Secretory epithelial cells (56.2%) and Perivascular cells (13.5%); the largest tumor fraction is Epithelial cells (52.7%) (Figure 2D). Median UMI counts across labeled cell types span 3102–24539; point area encodes the actual number of retained nuclei (Figure 2E). Contours are estimated separately from the two observed libraries and are descriptive because only one library is available for each condition (Figure 2F).

**FIGURE 2:**
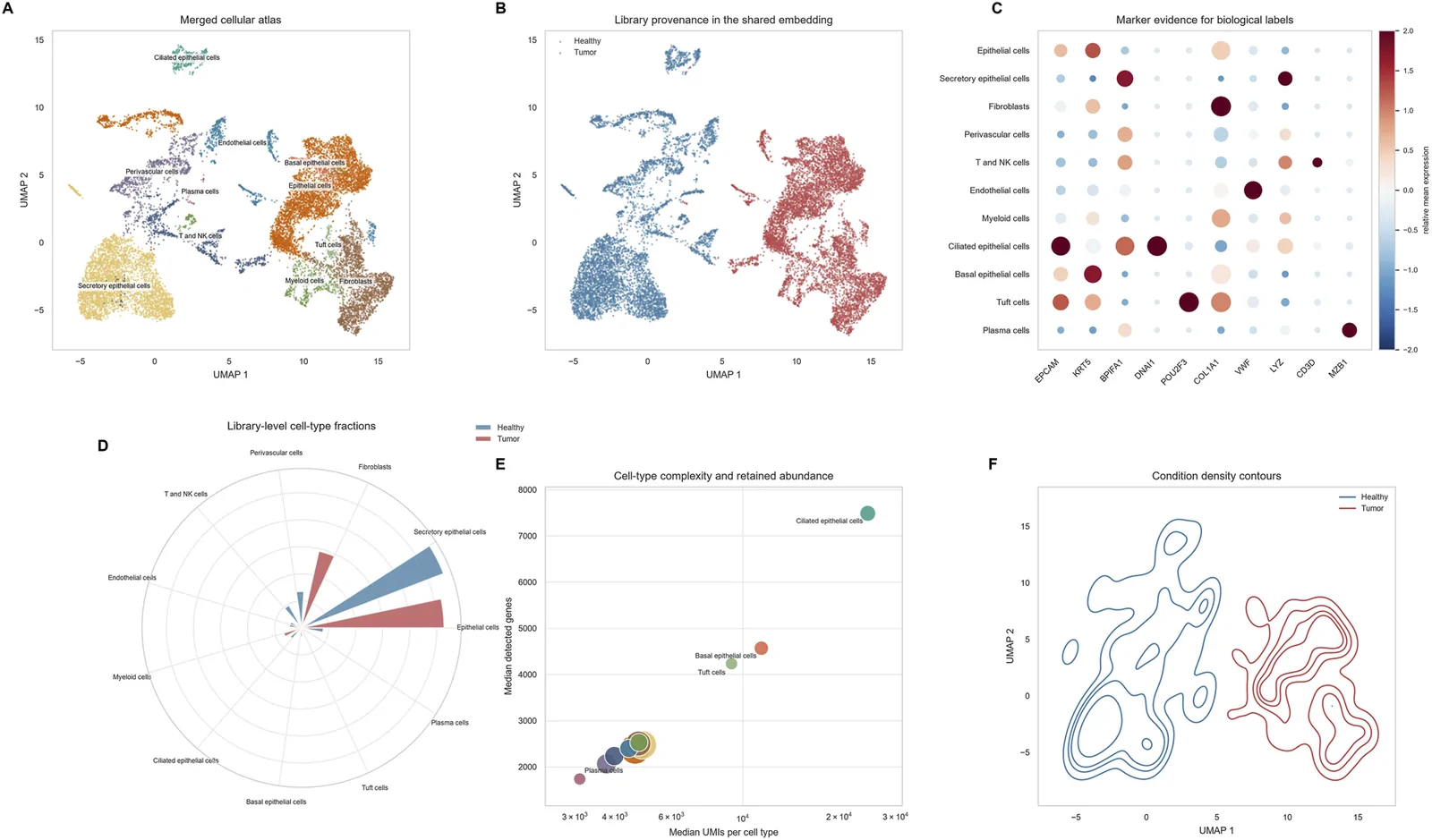
Merged single-nucleus cellular atlas. **(A)** The merged UMAP contains 14,479 retained nuclei annotated into 11 biological cell types: Epithelial cells, Secretory epithelial cells, Fibroblasts, Perivascular cells, T and NK cells, Endothelial cells, Myeloid cells, Ciliated epithelial cells, Basal epithelial cells, Tuft cells, Plasma cells. **(B)** HC1 contributes 6,817 healthy nuclei and CA5 contributes 7,662 tumor nuclei; this panel displays library provenance rather than a patient-level comparison. **(C)** The dot matrix uses observed normalized expression and detection fractions for 10 canonical genes across 11 labeled cell types. **(D)** The highest healthy fractions are Secretory epithelial cells (56.2%) and Perivascular cells (13.5%); the largest tumor fraction is Epithelial cells (52.7%). **(E)** Median UMI counts across labeled cell types span 3102–24539; point area encodes the actual number of retained nuclei. **(F)** Contours are estimated separately from the two observed libraries and are descriptive because only one library is available for each condition.

### 3.3 Descriptive remodeling of cellular composition

Stacked fractions use every retained nucleus in each library: 6,817 healthy and 7,662 tumor nuclei (Figure 3A). The largest observed increase is Epithelial cells (+44.7 percentage points), while the largest decrease is Secretory epithelial cells (−56.2 percentage points) (Figure 3B). Rank-abundance profiles display all 11 labeled cell types; the leading fractions are 56.2% in healthy tissue and 52.7% in tumor tissue (Figure 3C). The polar display repeats the observed library-level fractions without making a cell-state transition claim (Figure 3D). The concentration curves are based on 11 labeled cell types and describe within-library abundance inequality (Figure 3E). The lollipop display shows each cell type’s -p log(p) contribution; contributions sum to Shannon diversity values of 1.444 for the healthy library and 1.285 for the tumor library (Figure 3F).

**FIGURE 3:**
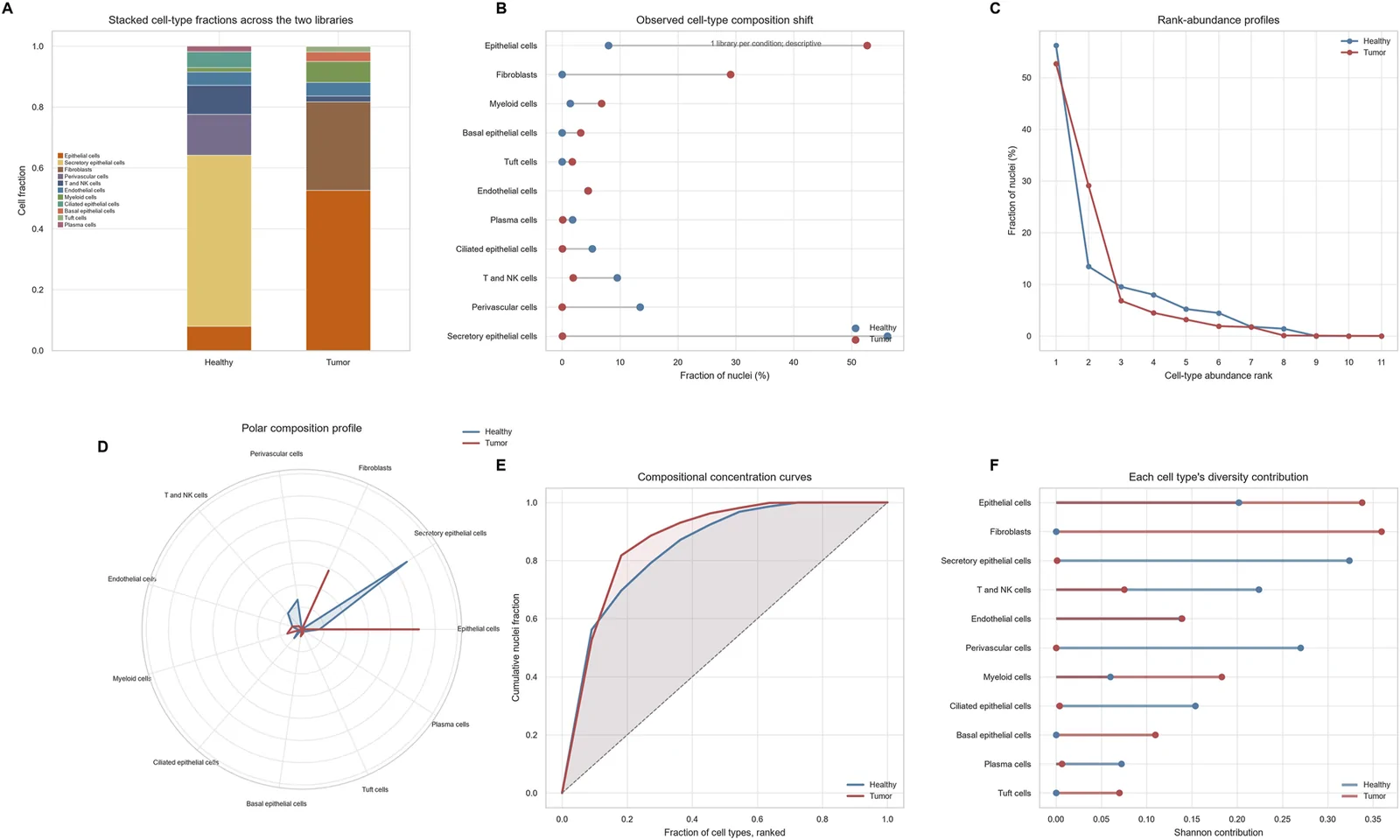
Descriptive remodeling of cellular composition. **(A)** Stacked fractions use every retained nucleus in each library: 6,817 healthy and 7,662 tumor nuclei. **(B)** The largest observed increase is Epithelial cells (+44.7 percentage points), while the largest decrease is Secretory epithelial cells (−56.2 percentage points). **(C)** Rank-abundance profiles display all 11 labeled cell types; the leading fractions are 56.2% in healthy tissue and 52.7% in tumor tissue. **(D)** The polar display repeats the observed library-level fractions without making a cell-state transition claim. **(E)** The concentration curves are based on 11 labeled cell types and describe within-library abundance inequality. **(F)** The lollipop display shows each cell type’s -p log(p) contribution; contributions sum to Shannon diversity values of 1.444 for the healthy library and 1.285 for the tumor library.

### 3.4 Cellular programs in the single-nucleus reference

Basal epithelial scores are calculated from 5 pre-specified genes on the retained single-nucleus object (Figure 4A). Median interferon-response score is 0.000 in healthy and 0.000 in tumor nuclei; individual cells are not treated as independent patients (Figure 4B). The bubble matrix summarizes nine observed programs across 11 biological cell types; color is relative mean score and size is the nonzero-score fraction (Figure 4C). The program network displays the 12 strongest observed Spearman edges across cell-type average scores (Figure 4D). Hypoxia-glycolysis and interferon-response scores have cell-level Spearman rho = −0.025 (computed across 14,479 retained nuclei and reported descriptively) (Figure 4E). The nine program-level median observed log2 CPM shifts are calculated from the two available libraries and summarize 45 observed gene-program values (Figure 4F).

**FIGURE 4:**
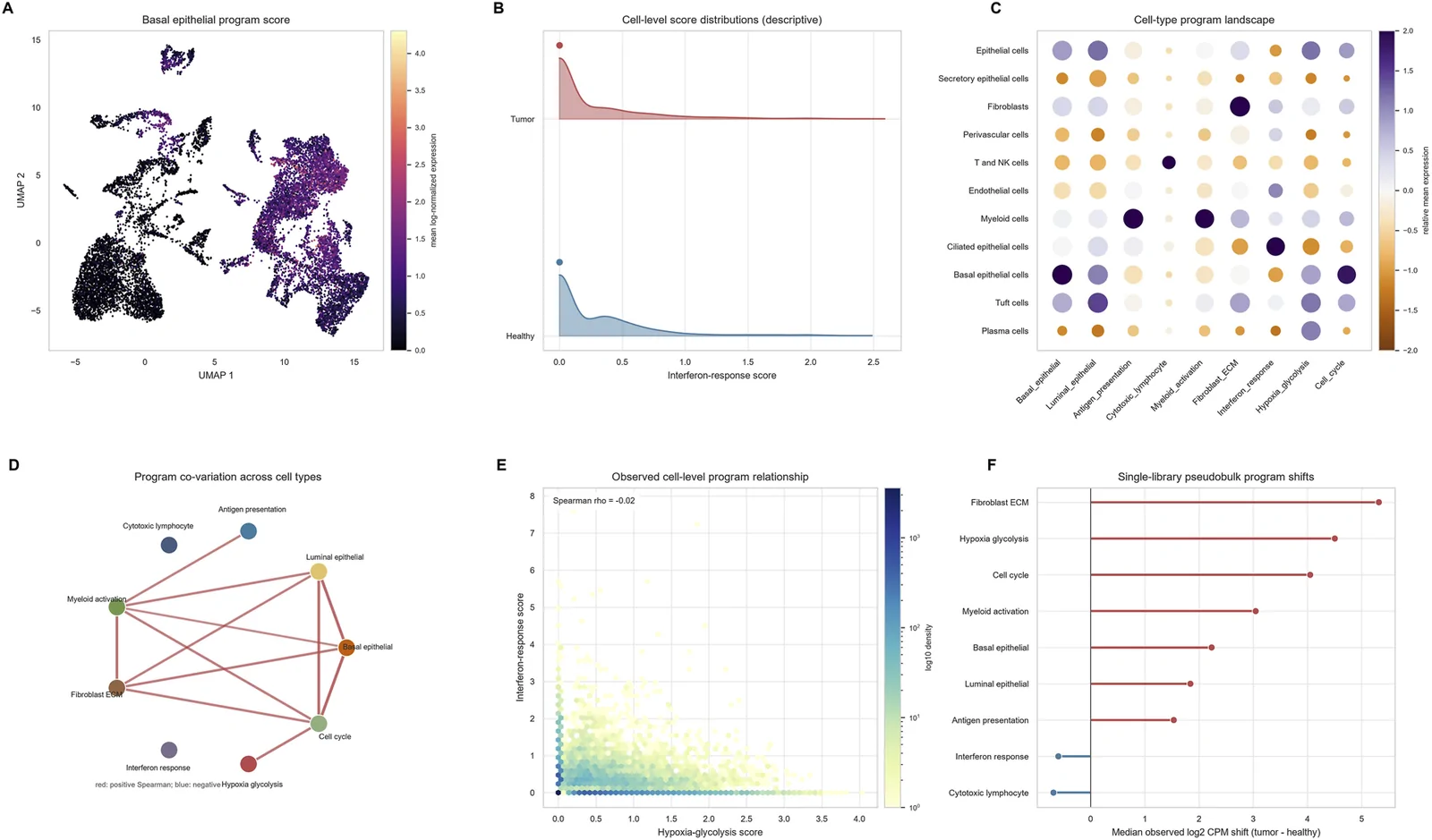
Cellular programs in the single-nucleus reference. **(A)** Basal epithelial scores are calculated from 5 pre-specified genes on the retained single-nucleus object. **(B)** Median interferon-response score is 0.000 in healthy and 0.000 in tumor nuclei; individual cells are not treated as independent patients. **(C)** The bubble matrix summarizes nine observed programs across 11 biological cell types; color is relative mean score and size is the nonzero-score fraction. **(D)** The program network displays the 12 strongest observed Spearman edges across cell-type average scores. **(E)** Hypoxia-glycolysis and interferon-response scores have cell-level Spearman rho = −0.025 (computed across 14,479 retained nuclei and reported descriptively). **(F)** The nine program-level median observed log2 CPM shifts are calculated from the two available libraries and summarize 45 observed gene-program values.

### 3.5 Bulk RNA-seq differential-expression landscape

PCA uses TMM logCPM expression for 14 GSE278145 samples (7 normal and seven tumor); PC1 and PC2 explain 44.7% and 15.4% of variance (Figure 5A). The sample-correlation matrix includes the same 14 bulk libraries after count-aware normalization (Figure 5B). The MA-style panel contains 17,677 tested genes and colors 5,732 genes with edgeR FDR <0.05 (Figure 5C). The hexbin volcano visualizes the full tested-gene distribution with the FDR = 0.05 reference line (Figure 5D). The lollipop panel shows six largest positive and six largest negative FDR-significant log2 fold changes, calculated as tumor minus normal (Figure 5E). Condition-wise density curves show the distribution of TMM logCPM values across the seven normal and seven tumor libraries; this is an expression-scale diagnostic rather than a differential-gene count panel (Figure 5F).

**FIGURE 5:**
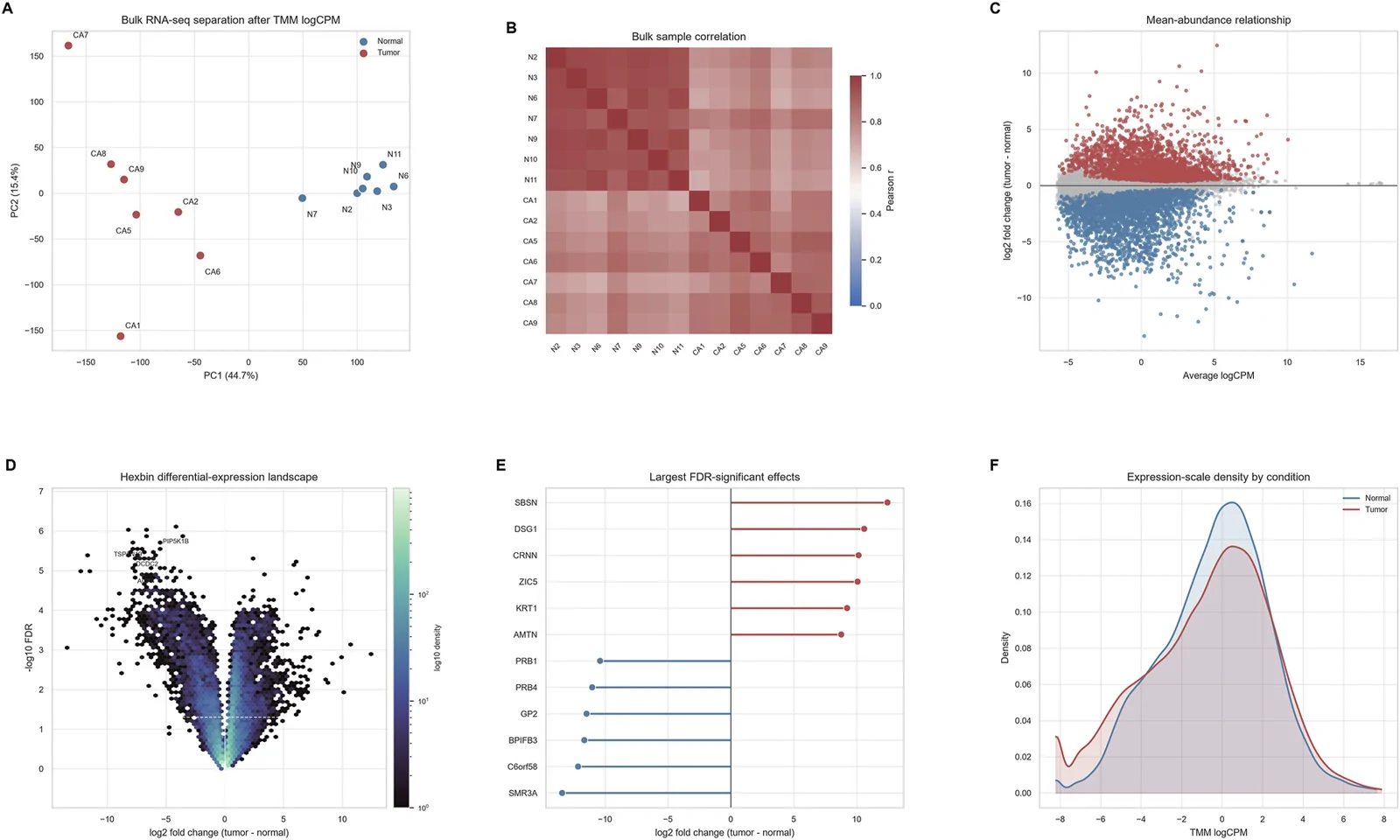
Bulk RNA-seq differential-expression landscape. **(A)** PCA uses TMM logCPM expression for 14 GSE278145 samples (7 normal and seven tumor); PC1 and PC2 explain 44.7% and 15.4% of variance. **(B)** The sample-correlation matrix includes the same 14 bulk libraries after count-aware normalization. **(C)** The MA-style panel contains 17,677 tested genes and colors 5,732 genes with edgeR FDR <0.05. **(D)** The hexbin volcano visualizes the full tested-gene distribution with the FDR = 0.05 reference line. **(E)** The lollipop panel shows six largest positive and six largest negative FDR-significant log2 fold changes, calculated as tumor minus normal. **(F)** Condition-wise density curves show the distribution of TMM logCPM values across the seven normal and seven tumor libraries; this is an expression-scale diagnostic rather than a differential-gene count panel.

#### 3.5.1 Bulk program structure and gene-set context

Running-enrichment curves use the complete edgeR tumor-versus-normal rank without simulated null curves; Interferon response: ES = 0.926, five observed genes; Myeloid activation: ES = −0.424, five observed genes; Fibroblast ECM: ES = 0.733, five observed genes (Figure 6A). The lollipop panel shows mean standardized program-score shifts across seven tumor versus seven normal libraries; the largest magnitude is Cell cycle (+1.727) (Figure 6B). Antigen-presentation score means are +0.238 in normal and −0.238 in tumor bulk samples (n = 7 per group) (Figure 6C). The bulk program network retains the 12 strongest observed Spearman correlations among the nine program scores (Figure 6D). The panel displays 11 genes drawn from 4 pre-specified programs, retaining up to three lowest-FDR measured members per program with FDR <0.05; node color reflects the observed log2 fold-change direction (Figure 6E). Ridgelines use observed log2 fold changes from 45 gene-program entries across nine gene sets (Figure 6F).

**FIGURE 6:**
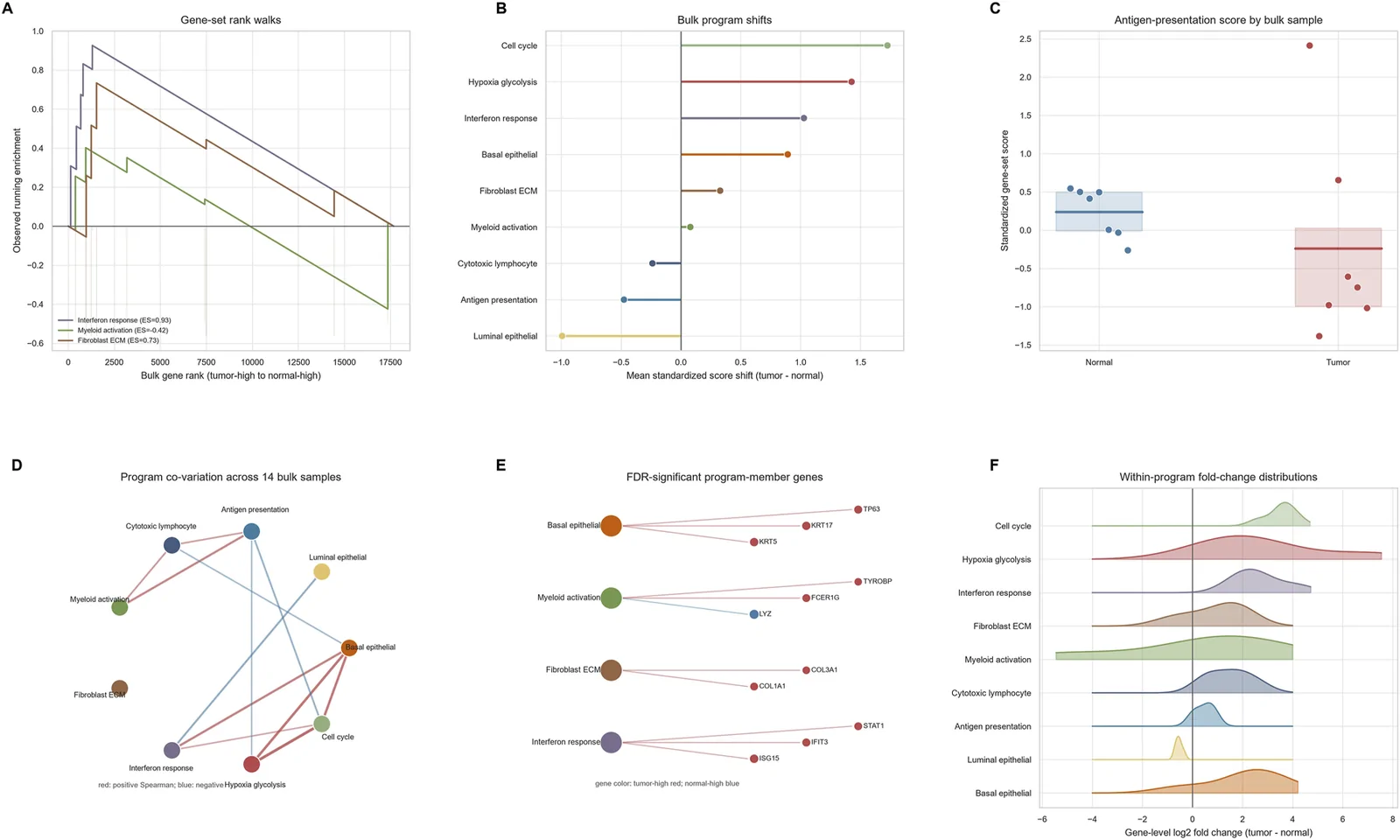
Bulk program structure and gene-set context. **(A)** Running-enrichment curves use the complete edgeR tumor-versus-normal rank without simulated null curves; Interferon response: ES = 0.926, five observed genes; Myeloid activation: ES = −0.424, five observed genes; Fibroblast ECM: ES = 0.733, five observed genes. **(B)** The lollipop panel shows mean standardized program-score shifts across seven tumor versus seven normal libraries; the largest magnitude is Cell cycle (+1.727). **(C)** Antigen-presentation score means are +0.238 in normal and −0.238 in tumor bulk samples (n = 7 per group). **(D)** The bulk program network retains the 12 strongest observed Spearman correlations among the nine program scores. **(E)** The panel displays 11 genes drawn from 4 pre-specified programs, retaining up to three lowest-FDR measured members per program with FDR <0.05; node color reflects the observed log2 fold-change direction. **(F)** Ridgelines use observed log2 fold changes from 45 gene-program entries across nine gene sets.

### 3.6 Paired and tumor-only public-cohort context within sinonasal squamous disease

GSE138809 contains five matched SNSCC tumor-adjacent-normal pairs; pair lines in the PCA connect the observed matched samples only (Figure 7A). Six probes with the smallest paired limma adjusted P values are shown across all five real pairs; probe identifiers are retained because this platform’s annotation cannot support gene-level mapping (Figure 7B). The paired limma model tested 60,755 probes and identified 2,620 probe-level features at adjusted P < 0.05 (Figure 7C). The GSE118386 context panel contains 21 NKSCC tumor-only samples; it is not used as a tumor-normal validation cohort (Figure 7D). Ridge distributions are computed from 7 pre-specified programs in those 21 tumor-only NKSCC samples (Figure 7E). The lollipop panel shows 8 FDR-ranked members selected from pre-specified programs, with sufficient expression and exact matches in the NKSCC tumor-only matrix; values are medians, not effect-size replication tests (Figure 7F).

**FIGURE 7:**
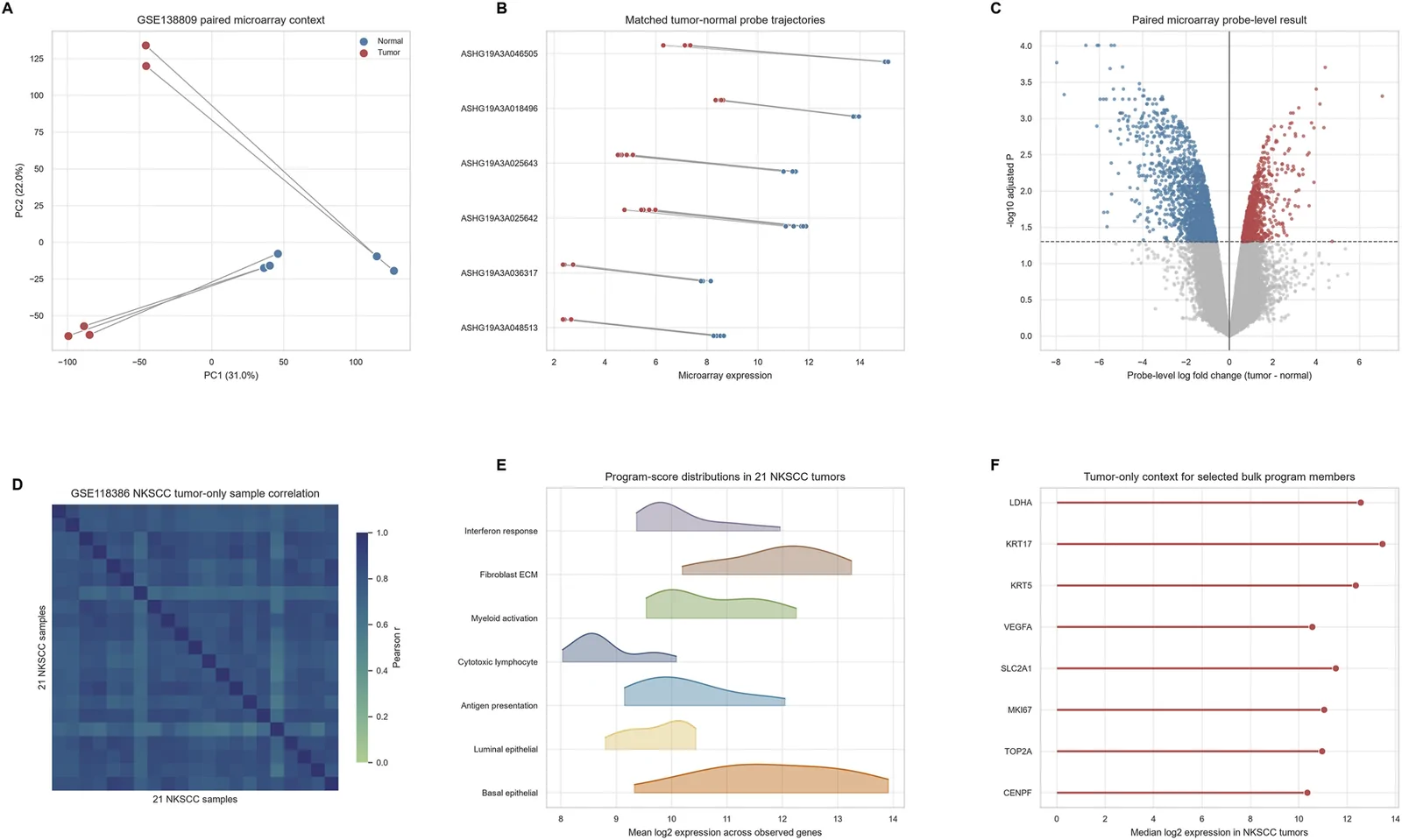
Paired and tumor-only public-cohort context within sinonasal squamous disease. **(A)** GSE138809 contains five matched SNSCC tumor-adjacent-normal pairs; pair lines in the PCA connect the observed matched samples only. **(B)** Six probes with the smallest paired limma adjusted P values are shown across all five real pairs; probe identifiers are retained because this platform’s annotation cannot support gene-level mapping. **(C)** The paired limma model tested 60,755 probes and identified 2,620 probe-level features at adjusted P < 0.05. **(D)** The GSE118386 context panel contains 21 NKSCC tumor-only samples; it is not used as a tumor-normal validation cohort. **(E)** Ridge distributions are computed from 7 pre-specified programs in those 21 tumor-only NKSCC samples. **(F)** The lollipop panel shows 8 FDR-ranked members selected from pre-specified programs, with sufficient expression and exact matches in the NKSCC tumor-only matrix; values are medians, not effect-size replication tests.

### 3.7 Cross-layer concordance and tumor-only context

Exact gene-symbol matching yielded 16,148 genes with both bulk edgeR effects and single-library observed pseudobulk shifts; Spearman rho = 0.543 (Figure 8A). The bulk set contains 5,732 FDR<0.05 genes. The single-nucleus candidate set is the empirical top 10% of absolute observed shifts (cutoff 2.865), yielding 1,615 genes and 975 shared genes (Figure 8B). The panel displays nine genes selected from pre-specified program membership by combined observed effect size after bulk FDR<0.05, logCPM>1, and concordant single-library direction filters; it is exploratory, not independent replication testing (Figure 8C). Cell-type bubbles are calculated from the observed normalized expression and detection fraction of eight selected candidate genes (Figure 8D). Across 9 pre-specified programs, the cross-scale Spearman rho is 0.467; the single-nucleus axis remains descriptive because it compares one healthy and one tumor library (Figure 8E). Nine selected candidates have exact gene-symbol matches in the 21-sample NKSCC tumor-only matrix; medians and Q1-Q3 intervals provide context only and do not constitute a tumor-normal validation test (Figure 8F).

**FIGURE 8:**
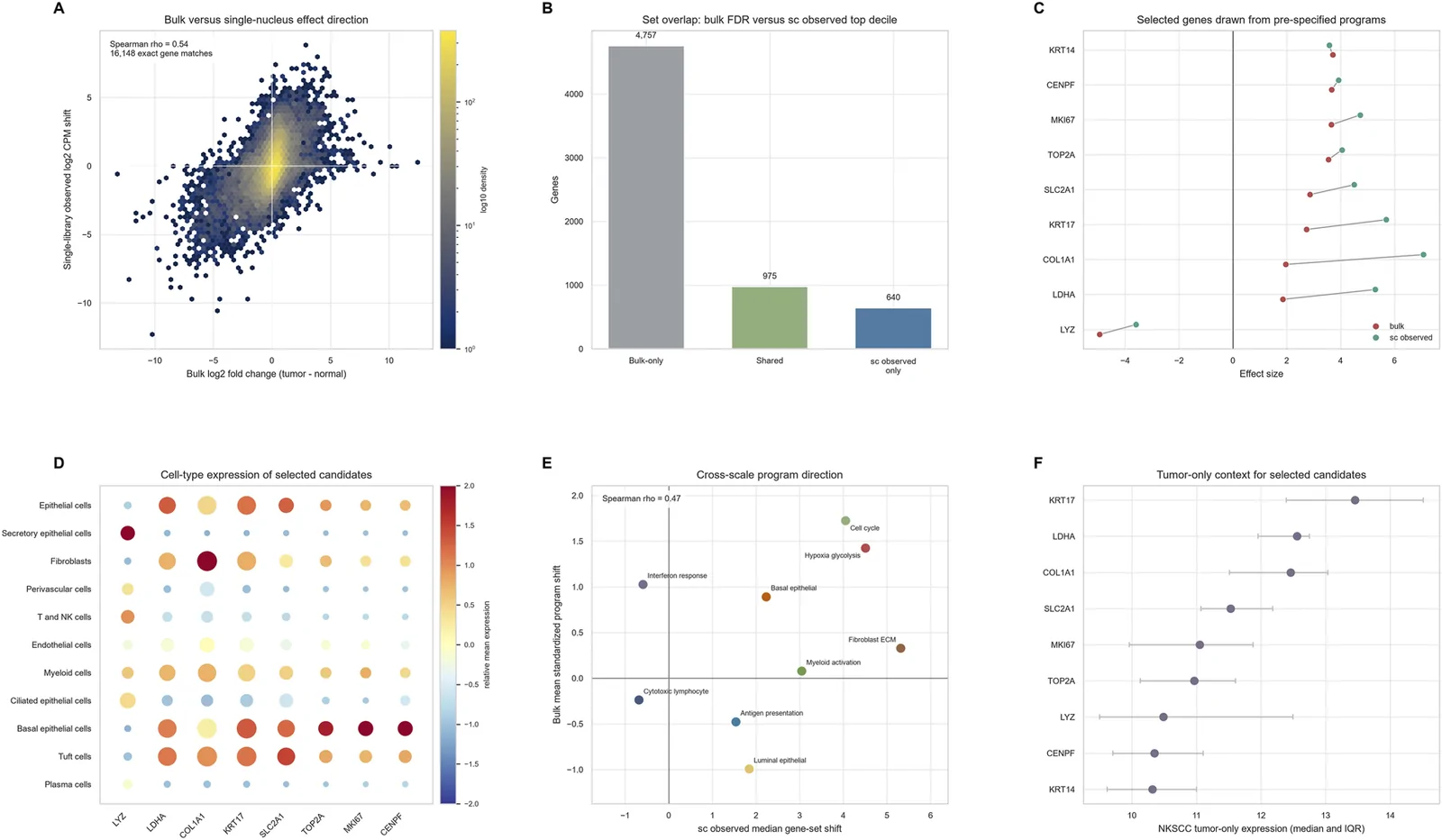
Cross-layer concordance and tumor-only context. **(A)** Exact gene-symbol matching yielded 16,148 genes with both bulk edgeR effects and single-library observed pseudobulk shifts; Spearman rho = 0.543. **(B)** The bulk set contains 5,732 FDR<0.05 genes. The single-nucleus candidate set is the empirical top 10% of absolute observed shifts (cutoff 2.865), yielding 1,615 genes and 975 shared genes. **(C)** The panel displays nine genes selected from pre-specified program membership by combined observed effect size after bulk FDR<0.05, logCPM>1, and concordant single-library direction filters; it is exploratory, not independent replication testing. **(D)** Cell-type bubbles are calculated from the observed normalized expression and detection fraction of eight selected candidate genes. **(E)** Across 9 pre-specified programs, the cross-scale Spearman rho is 0.467; the single-nucleus axis remains descriptive because it compares one healthy and one tumor library. **(F)** Nine selected candidates have exact gene-symbol matches in the 21-sample NKSCC tumor-only matrix; medians and Q1-Q3 intervals provide context only and do not constitute a tumor-normal validation test.

### 3.8 In Vitro validation of candidate gene expression in SNSCC cell lines by qRT-PCR and ELISA

To provide preliminary *in vitro* evidence for the tumor-versus-normal upregulation of candidate genes identified in the transcriptomic analysis, we performed qRT-PCR and ELISA assays in two SNSCC cell lines (RPMI 2650 and Detroit 562) compared with the normal bronchial epithelial cell line BEAS-2B. These experiments constitute a cancer-versus-normal comparison and do not address HPV-subtype differences; neither RPMI 2650 nor Detroit 562 is an established HPV-positive model. qRT-PCR analysis demonstrated significant mRNA upregulation of all four candidate genes in SNSCC cell lines (Figure 9A). In RPMI 2650 cells, CD74, CCL5, IL32, and HLA-DRA showed 4.27 ± 0.38-, 3.15 ± 0.27-, 3.68 ± 0.31-, and 3.82 ± 0.33-fold increases, respectively (all p < 0.01). Detroit 562 cells exhibited similar but comparatively lower overexpression (CD74: 3.53 ± 0.30-fold; CCL5: 2.46 ± 0.21-fold; IL32: 2.91 ± 0.25-fold; HLA-DRA: 3.14 ± 0.27-fold; all p < 0.01), potentially reflecting differences in tissue origin between the 2 cell lines. ELISA further corroborated these findings at the protein level (Figure 9B). Protein concentrations of all four biomarkers were significantly elevated in both SNSCC lines relative to BEAS-2B (all p < 0.01). CD74 showed the most pronounced upregulation, with a 4.68-fold protein increase in RPMI 2650 cells (p < 0.001), consistent with its critical role as the MHC class II invariant chain in antigen presentation and adaptive immune activation. These findings provide preliminary *in vitro* evidence that the candidate genes CD74, CCL5, IL32, and HLA-DRA are upregulated in SNSCC epithelial cells relative to normal bronchial epithelium, consistent with the bulk transcriptomic findings. Whether this upregulation extends to HPV-subtype differences or predicts immunotherapy sensitivity requires validation in appropriate HPV-matched models and clinical cohorts.

**FIGURE 9:**
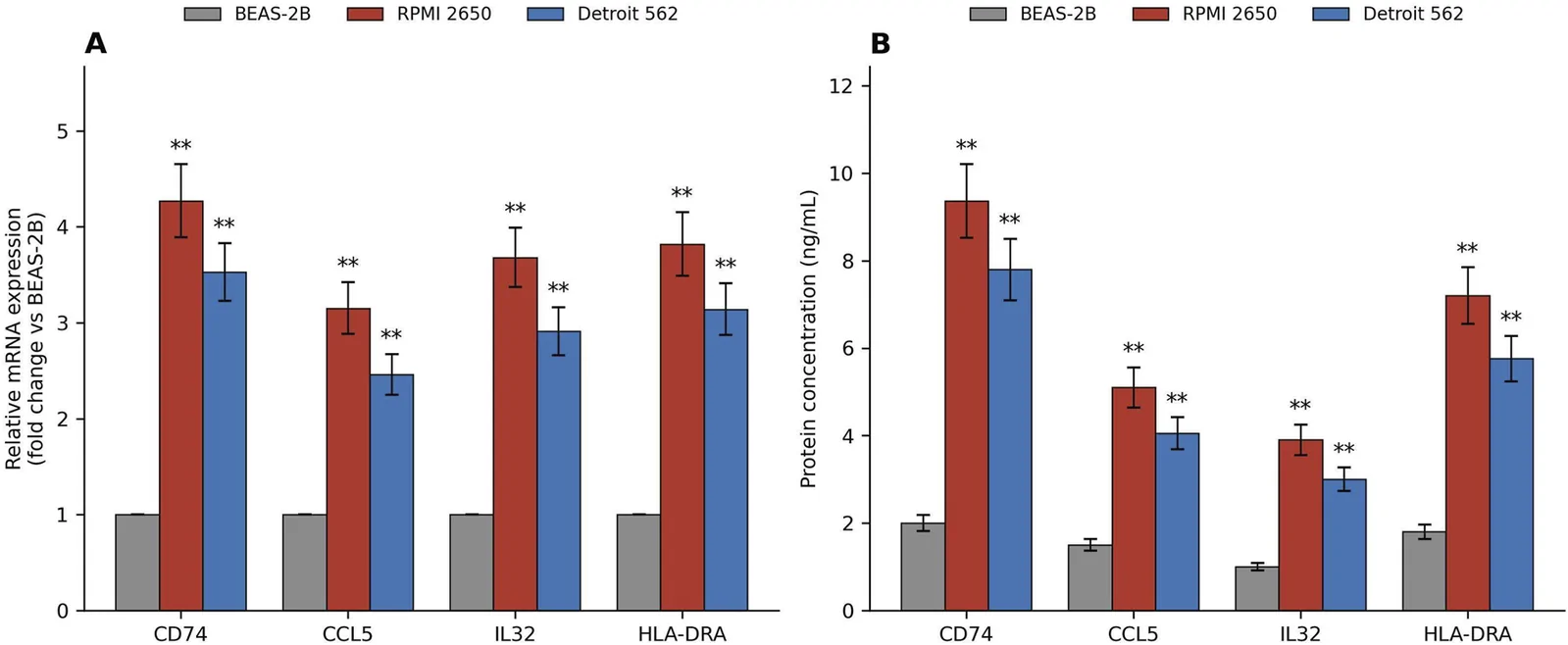
*In vitro* validation of candidate gene expression in SNSCC cell lines by qRT-PCR and ELISA. **(A)** qRT-PCR analysis of relative mRNA expression levels of CD74, CCL5, IL32, and HLA-DRA in SNSCC cell lines (RPMI 2650 and Detroit 562) compared to normal human bronchial epithelial BEAS-2B cells. Expression values are presented as fold change relative to BEAS-2B (set as 1.0), normalized to GAPDH. **(B)** ELISA quantification of protein concentrations (ng/mL) of CD74, CCL5, IL32, and HLA-DRA in cell lysates from BEAS-2B, RPMI 2650, and Detroit 562 cells. Data are presented as mean ± SEM from three independent experiments performed in triplicate. **p < 0.01 compared to BEAS-2B by one-way ANOVA with Tukey’s post-hoc test.

## 4 Discussion

This multi-layer transcriptomic reanalysis of publicly available SNSCC datasets provides descriptive insights into tumor-versus-normal cellular and molecular remodeling. Because the single-nucleus data are derived from one healthy library (HC1) and one tumor library (CA5), observations comparing these two libraries reflect individual library-level differences and cannot be attributed to HPV status or generalized to the SNSCC population. The bulk RNA-seq findings, based on seven tumor and seven normal samples from GSE278145, provide a more robust estimation of tumor-versus-normal differential expression and are the primary evidence layer for candidate gene nomination.

The observed compositional shift between the healthy (HC1) and tumor (CA5) single-nucleus libraries—most notably an increase in Epithelial cells (+44.7 percentage points) and a corresponding decrease in Secretory epithelial cells (−56.2 percentage points)—is consistent with the known propensity of SNSCC to expand undifferentiated epithelial populations at the expense of specialized secretory lineages. Because only one library per condition is available, these compositional observations are descriptive and cannot be attributed to HPV status, patient-level factors, or generalized to the broader SNSCC population (Mandal et al., 2016).

The expression patterns of key marker genes provide mechanistic insights into immune microenvironment function. CD74, encoding the MHC class II invariant chain essential for antigen presentation, was among the top FDR-significant upregulated genes in the bulk RNA-seq analysis and showed widespread expression across immune cell populations in the single-nucleus data with Myeloid cell enrichment. The co-expression of CD74 with other MHC class II components (HLA-DRA, HLA-DRB1) suggests coordinated upregulation of these individual antigen-presentation genes in the SNSCC microenvironment; notably, this gene-level pattern occurred alongside a lower composite antigen-presentation program score in tumor relative to normal bulk samples (Figure 6C), suggesting gene-specific rather than program-wide induction. Upregulation of individual MHC class II genes has been associated with favorable prognosis and immunotherapy response in other cancer types (Roche and Furuta, 2015; Johnson et al., 2016). Importantly, integrative transcriptomic approaches that combine multi-layer data—including Mendelian randomization and bulk RNA-seq—have proven valuable in disentangling genetic from environmental contributions to disease-relevant gene expression programs (Zhang et al., 2025), providing a methodological precedent for the cross-platform integration strategy employed in the present study.

CCL5 (RANTES), a critical chemokine mediating recruitment of T cells, NK cells, and other immune effectors, exhibited concentrated expression in specific immune-active clusters. CCL5 plays roles in orchestrating adaptive immune responses and has been associated with immune infiltration and outcomes in head and neck cancers (Albers et al., 2005). In the bulk RNA-seq data, CCL5 shows FDR-significant upregulation in tumor versus normal samples, and its cross-layer concordance with single-nucleus observations supports its nomination as a candidate biomarker.

IL32, a potent proinflammatory cytokine produced by activated T cells and NK cells, showed cluster-specific expression patterns consistent with immune activation signatures. IL32 has been implicated in antitumor immunity through multiple mechanisms including NK cell activation, dendritic cell maturation, and direct cytotoxic effects on tumor cells (Kim et al., 2005). The identification of IL32 shows concordant upregulation across bulk and single-nucleus data layers, supporting its nomination as a candidate biomarker. Prospective studies are needed to determine whether IL32 expression levels are associated with immunotherapy response in SNSCC.

The bulk RNA-seq differential expression analysis identifying MHC class II genes and interferon-stimulated genes among the top FDR-significant genes provides molecular evidence for immunologically active programs in SNSCC tumors. Enrichment of interferon response genes (ES = 0.926) in the tumor-versus-normal comparison is consistent with active innate immune activation in the tumor microenvironment. Interferon-gamma signaling has been associated with response to checkpoint blockade immunotherapy across multiple cancer types (Ayers et al., 2017). Whether the interferon program enrichment observed here translates to immunotherapy sensitivity in SNSCC requires prospective clinical validation.

The program connectivity analysis using PAGA-based graph structure reveals associations among cell-type average program scores within the single-nucleus object. These graph-level connections reflect co-occurrence and correlation patterns across the two observed libraries and should not be interpreted as directed differentiation relationships or lineage hierarchies across distinct biological lineages such as epithelial, myeloid, and lymphoid compartments.

The immune cell program scoring analysis demonstrates heterogeneous T cell, B cell, and macrophage program activity across the 11 annotated cell types within the single-nucleus object. These observations are descriptive and reflect single-library program scores; they cannot be extrapolated to architectural features such as tertiary lymphoid structures without spatial validation (Helmink et al., 2020).

Several limitations of this study warrant consideration. First, the single-nucleus data consist of one healthy library (HC1) and one tumor library (CA5), and any library-level differences cannot be attributed to HPV status or generalized to the SNSCC population; independent replication in additional samples is required before any comparative claim can be made. Second, HPV status annotations for the single-nucleus libraries were derived from the deposited metadata of the original GEO submissions; no independent viral genome mapping, E6/E7 transcript detection, or p16 immunostaining was performed. Third, the gene signatures used for immune cell scoring were computed at the level of major immune lineages; finer-grained subpopulation scoring (e.g., CD4^+^ T, CD8^+^ effector/memory T, Treg, plasma B, memory B, macrophage, dendritic cell, and MDSC) would provide greater biological resolution and is a priority for future reanalysis with additional samples. Fourth, spatial and tissue-level validation by IHC or immunofluorescence staining for CD74, CCL5, IL32, and HLA-DRA in an independent SNSCC tissue microarray cohort is needed to confirm the cell-type origin and spatial distribution of the identified signals. Fifth, cross-referencing the candidate biomarkers identified here against the CIDE framework (Gong et al., Cell, 2025), which integrates 90 immunotherapy omics datasets including head and neck cancers, would strengthen assessment of their association with immune checkpoint blockade response and represents a high-priority direction for future work. Sixth, ambient RNA contamination was not estimated because empty-droplet matrices were not supplied with the deposited data; enrichment of ribosomal protein genes (e.g., RPS12) in marker lists may partly reflect this technical artifact. Finally, the *in vitro* experiments compare SNSCC cell lines against normal bronchial epithelium and therefore constitute a cancer-versus-normal comparison rather than an HPV-subtype comparison; no immunotherapy treatment or response outcome data were assessed, and all nominated genes should be considered candidate biomarkers pending prospective clinical validation.

Despite these limitations, this study provides an integrative descriptive framework for tumor-versus-normal transcriptomic remodeling in SNSCC using publicly available multi-layer data. The nomination of CD74, CCL5, IL32, and HLA-DRA as candidate biomarkers is supported by cross-layer concordance across single-nucleus and bulk RNA-seq platforms, and by preliminary *in vitro* evidence of upregulation in SNSCC epithelial cells. These candidates warrant prospective validation in larger independent cohorts with HPV-matched tissue specimens, IHC confirmation, and—where feasible—immunotherapy outcome data.

## 5 Conclusion

In conclusion, this multi-layer transcriptomic reanalysis of publicly available SNSCC datasets provides descriptive evidence for widespread tumor-versus-normal gene expression dysregulation and identifies a cross-layer cell-cycle and immune-program signal. CD74, CCL5, IL32, and HLA-DRA emerge as candidate biomarkers with concordant upregulation across single-nucleus and bulk transcriptomic layers and preliminary *in vitro* support. All findings are exploratory; HPV-subtype-specific inferences are not supported by the available single-sample single-nucleus data and require validation in larger, appropriately powered, HPV-matched cohorts.

## Data Availability

The original contributions presented in the study are publicly available. This data can be found in the NCBI GEO repository with the accession numbers GSE277797 (RNA components GSM8530826/HC1 and GSM8530830/CA5), GSE278145, GSE138809, and the analyzed 21-sample NKSCC subset of GSE118386. Further inquiries can be directed to the corresponding author.
